# High Throughput Screening of a Prescription Drug Library for Inhibitors of Organic Cation Transporter 3, OCT3

**DOI:** 10.1007/s11095-022-03171-8

**Published:** 2022-01-28

**Authors:** Eugene C. Chen, Pär Matsson, Mina Azimi, Xujia Zhou, Niklas Handin, Sook Wah Yee, Per Artursson, Kathleen M. Giacomini

**Affiliations:** 1 Department of Drug Metabolism and Pharmacokinetics, Genentech, Inc., South San Francisco, California USA; 2 Department of Pharmacology, Sahlgrenska Academy, University of Gothenburg, Gothenburg, Sweden; 3 Department of Pharmacokinetics, Pharmacodynamics and Drug Metabolism, Merck & Co., Kenilworth, NJ USA; 4 Department of Bioengineering and Therapeutic Sciences, University of California, San Francisco, San Francisco, California USA; 5 Department of Pharmacy and Science for Life Laboratory, Uppsala University, Uppsala, Sweden

**Keywords:** Solute carrier superfamily, extraneuronal monoamine transporter, EMT

## Abstract

**Introduction:**

The organic cation transporter 3 (OCT3, SLC22A3) is ubiquitously expressed and interacts with a wide array of compounds including endogenous molecules, environmental toxins and prescription drugs. Understudied as a determinant of pharmacokinetics and pharmacodynamics, OCT3 has the potential to be a major determinant of drug absorption and disposition and to be a target for drug-drug interactions (DDIs).

**Goal:**

The goal of the current study was to identify prescription drug inhibitors of OCT3.

**Methods:**

We screened a compound library consisting of 2556 prescription drugs, bioactive molecules, and natural products using a high throughput assay in HEK-293 cells stably expressing OCT3.

**Results:**

We identified 210 compounds that at 20 μM inhibit 50% or more of OCT3-mediated uptake of 4-Di-1-ASP (2 μM). Of these, nine were predicted to inhibit the transporter at clinically relevant unbound plasma concentrations. A Structure-Activity Relationship (SAR) model included molecular descriptors that could discriminate between inhibitors and non-inhibitors of OCT3 and was used to identify additional OCT3 inhibitors. Proteomics of human brain microvessels (BMVs) indicated that OCT3 is the highest expressed OCT in the human blood-brain barrier (BBB).

**Conclusions:**

This study represents the largest screen to identify prescription drug inhibitors of OCT3. Several are sufficiently potent to inhibit the transporter at therapeutic unbound plasma levels, potentially leading to DDIs or off-target pharmacologic effects.

**Supplementary Information:**

The online version contains supplementary material available at 10.1007/s11095-022-03171-8.

## Introduction

The organic cation transporter 3 (OCT3), also known as extraneuronal monoamine transporter (EMT), is a member of the OCT subfamily of the SLC22 family of transporter proteins. Like its paralogs OCT1 and OCT2, OCT3 interacts with a wide array of endogenous and exogenous small organic cations, including vitamins, neurotransmitters, environmental chemicals and prescription drugs (1–3). However, unlike OCT1 and OCT2, which are predominantly expressed in the liver and kidney, respectively, OCT3 displays a more ubiquitous expression profile (4). Widely expressed in many tissues including placenta, heart, lung, liver, intestine, prostate, skeletal muscle, and brain and diverse substrates, OCT3 is involved in many pleiotropic effects. Notably, impaired function in OCT3 has been shown to have effects on cellular metabolism, energy production, cardiac function, and neuropsychiatric traits (5).

Human genetic studies have revealed important roles of OCT3 in metabolic and cardiac effects. For example, *OCT3* genetic variants have been found to be associated, at genomewide levels of significance, with interindividual differences in metabolic rates (6). Further, loss of the transporter in mouse adipocytes results in increased body temperature, thermogenesis, and lipid breakdown, as well as effects on mitochondrial biogenesis (6). Human genetic studies also reveal roles of OCT3 in cardiac pathologies including coronary artery disease, myocardial infarction, and ischemic stroke (7–10). In addition, OCT3 has been linked to various neuropsychiatric phenotypes (11). For example, low expression levels of OCT3 in the central nervous system (CNS) of mice are associated with behavioral changes reminiscent of reduced depression (12). Deficiency in the serotonin (5-HT) transporter, SERT, linked to mood disorders and depression, has been shown to be compensated for by increased OCT3 levels in the brains of mice (13). OCT3 also has a role in dopaminergic neurodegeneration related to exposure to the cationic neurotoxins, N-methylpyridinium (MPP^+^) and paraquat, which are substrates of the transporter (14, 15). In humans, mutations in *OCT3* have been linked to children with obsessive compulsive disorder (16).

Though its paralogs OCT1 and OCT2 have been shown to be major determinants of the pharmacokinetics and pharmacodynamics of many drugs (17–24), OCT3 remains highly understudied with only a limited number of studies examining its role in drug absorption and disposition and as a potential target for drug-drug interactions (DDIs). In one study in *Oct3*^*−/−*^ mice, the pharmacokinetics of the anti-diabetic drug metformin was shown to be significantly modulated by the deletion of the transporter, consistent with an important role of the transporter in the absorption and disposition of metformin in mice (25). Metformin pharmacodynamics were also altered in the knockout mice, reflecting changes in its accumulation in various tissues such as skeletal muscle and adipose tissue. In addition to metformin, other prescription drugs across various therapeutic classes are substrates of the transporter (26). These include the bronchodilator, fenoterol, the anti-migraine medication, sumatriptan, the antiviral drug, lamuvidine, and the muscle relaxant, trospium, suggesting that the transporter may play a role in the pharmacokinetics of many drugs.

During drug development, regulatory authorities suggest screening new drugs against about ten drug transporters that are major determinants of pharmacokinetics and targets for DDIs. OCT2 is one of these transporters and more recently screening new drugs against OCT1 has been suggested (26, 27). Similarly, some have suggested screening new drugs as inhibitors of OCT3 because of its potential role in drug disposition and response (26). Though OCT1 and OCT2 have been studied extensively and we have previously conducted prescription drug library screens of both transporters (24, 28, 29), to date, no study has systematically identified inhibitors of OCT3.

The goal of the current study was to screen a prescription drug library to identify inhibitors of OCT3. In particular, we were interested in identifying drugs that may interact with OCT3 at clinically relevant concentrations. We developed an in vitro high throughput screen (HTS) using the fluorescent substrate, 4-Di-1-ASP (ASP^+^) in a cell line stably expressing OCT3. Our HTS identified 210 inhibitors of OCT3 out of the 2556 structurally diverse prescription drugs, natural products, and bioactive molecules screened. Most of inhibitors identified were previously not known to inhibit OCT3. Twenty-three of the 210 inhibitors were potent inhibitors and could potentially cause clinically relevant DDIs based on their predicted IC_50_ and reported plasma C_MAX_ values. Using the data from the screen, a predictive structure-activity relationship (SAR) model was developed to discriminate inhibitors from non-inhibitors of OCT3. Finally, our proteomic studies suggested that of the OCT paralogs, OCT3 is the most abundantly expressed in the human blood-brain barrier (BBB), consistent with a role in CNS drug disposition.

## Materials and Methods

### Chemicals

The MicroSource Spectrum compound library (Gaylordsville, CT) was obtained through the Small Molecular Discovery Center at the University of California, San Francisco (San Francisco, CA). 4-Di-1-ASP, termed ASP^+^, was purchased from Molecular Probes (Grand Island, NY). All other chemicals were purchased from Sigma-Aldrich (St. Louis, MO). All cell culture media and supplements were purchased from Life Technologies (Carlsbad, CA) except fetal bovine serum, which was purchased from GE Healthcare Life Sciences (South Logan, UT).

### Cell Culture

In previous studies from our laboratory human embryonic kidney (HEK-293) cell line stably overexpressing OCT3 was established and functionally validated using known inhibitors and non-inhibitors of OCT3 (30, 31). The cells were maintained in Dulbecco’s Modified Eagle’s Medium (DMEM H-21) supplemented with 75 μg/ml of hygromycin B, penicillin (100 U/ml), streptomycin (100 mg/ml), and 10% fetal bovine serum in a humidified atmosphere with 5% CO_2_ at 37°C.

### In Vitro Uptake Studies

HEK-293 cells overexpressing OCT3 were seeded in black, clear bottom poly-D-lysine coated 96-well plates (Greiner Bio-One, Monroe, NC) and allowed to grow for 48 h until approximately 90% confluency. For uptake kinetics study, cells were incubated with Hanks’ balanced salt solution (HBSS, ThermoFisher, Waltham, MA) containing serial dilution of ASP^+^ for 2 min at 37°C. At the end of experiments, the media was aspirated and the cells were washed twice with ice-cold HBSS containing 50 μM corticosterone, as an inhibitor of OCT3 to avoid efflux of the accumulated substrate. The K_m_ and V_max_ were calculated by fitting a Michaelis-Menten relationship to the data. For time course study, cells were incubated with HBSS containing 2 μM ASP^+^ at 37°C. At various time points, the experiment was stopped as previously described. For IC_50_ determination, cells were incubated with HBSS containing 2 μM ASP^+^ or 1 μM metformin with 0.5 μCi/ml [^14^C]metformin, and serial dilution of inhibitors for 2 min at 37°C. Inhibition data were fitted with nonlinear regression with variable slope, and IC_50_ values were determined through a standard sigmoidal curve fit with variable slope in GraphPad Prism. The signal of ASP^+^ was measured using an Analyst AD plate reader (Molecular Devices, Sunnyvale, CA) with excitation and emission filters tuned at 485 and 585 nm wavelength, respectively. All statistical analysis and curve fitting were done using GraphPad Prism version 6 software (La Jolla, CA).

### High Throughput Screening

The high throughput screen was performed at the Small Molecule Discovery Center at the University of California, San Francisco. HEK-293 cells overexpressing OCT3 were seeded in black, clear bottom poly-D-lysine coated 96-well plates (Greiner Bio-One, Monroe, NC) and allowed to grow for 48 h until approximately 90% confluency. Cells were incubated with HBSS containing 2 μM ASP^+^ and 20 μM of test compounds at ambient temperature for approximately 2 min. At the end of the experiment, media were aspirated and cells were washed twice with HBSS containing 50 μM corticosterone. Nonspecific transport was determined in wells on each assay plate using 100 μM corticosterone as OCT3 inhibitor. The screen was carried out with a Biomek FXp liquid handler (Beckman Coulter, Brea, CA). Fluorescence was measured as previously described. Predicted IC_50_ values were calculated using the following equation. IC_50_ for compounds that inhibit 80% or more at 20 μM were estimated to be 5 μM.

$$ Predicted\ {IC}_{50}=\left(\frac{100\%}{Percent\ Activity\ Inhibited}-1\right)\ast 20\ \upmu \mathrm{M} $$

### Molecular Descriptor Generation

The molecular descriptor generation was performed as previously described (28). Three-dimensional molecular structures were generated from SMILES representations using Corina, version 3.0 (Molecular Networks, Erlangen, Germany), keeping the lowest energy conformation of a maximum of 100 alternative ring conformations, and were used as input for molecular descriptor calculation with DragonX, version 1.4 (Talete, Milan, Italy), ADMETPredictor, version 5.0 (SimulationsPlus, Lancaster, CA), and MAREA, version 3.02 (32). After removal of replicate molecular descriptors and descriptors having zero variance, the remaining descriptors were used as the starting point for structure-activity model development.

### Structure-Activity Modeling

The structure-activity modeling was generated as previously described (28). Partial least-squares discriminant analysis (PLS-DA) was used to develop computational models that differentiate between OCT3 inhibitors and noninhibitors based on differences in molecular descriptor values. A double-loop cross-validation (CV) procedure was used to provide an unbiased estimate of the prediction accuracy: model optimization was performed in a ten-fold inner CV loop, estimating model improvement based on the withheld data, and the prediction accuracy of the optimized models were estimated from the withheld data in the outer CV loop. Through this double-loop procedure, model optimization and predictivity assessments were both based on data not used to train the model. In the inner CV loop, variable selection was performed in two phases: first, the descriptors with lowest absolute PLS weight were iteratively removed until only the 25 most important ones remained; second, the same procedure was repeated, but descriptors were kept in the model if removal resulted in an inferior model. The entire double-loop procedure was repeated 100 times for different random partitioning of the data set to enable calculation of confidence intervals of prediction accuracy estimates and model parameters. A skew-normal density function was fitted to the PLS prediction scores obtained from retrospective application of the final model to the HTS dataset, in order to transform the raw PLS scores to a probability of belonging to the inhibitor or the noninhibitor class. The resulting class probability function was then applied in prospective predictions of registered drugs in the DrugBank database (www.drugbank.ca).

### Human Brain Tissue Samples

Five healthy human post-mortem frozen brain cortical tissue samples (donors aged >16 years old) were obtained from the National Institutes of Health NeuroBioBank at the University of Maryland, Baltimore, MD. Tissues were stored at −80°C until day of microvessel isolation.

### Isolation of Human Brain Microvessels

Brain microvessels (BMVs) were isolated following a previously described protocol (33), with some modifications. All steps were carried out on ice or at 4°C starting with <1 g of brain cortical tissue. Samples were thawed and homogenized in HBSS containing protease inhibitors (cOmplete protease inhibitor cocktail, Sigma-Aldrich, St. Louis, MO) with 20 up-and-down strokes in a Potter-Elvehjem glass homogenizer. The homogenate was centrifuged at 1200 g for 10 min at 4°C, and the supernatant discarded. The resulting pellet enriched with BMVs was resuspended in a 17.5% dextran-70/HBSS solution and centrifuged at 4300 g for 15 min at 4°C in a swinging bucket rotor. The supernatant containing a myelin-rich layer was aspirated and the pellet resuspended in HBSS with 1% Bovine Serum Albumin (BSA). This solution was passed through a 40 μm nylon mesh filter and the BMVs captured on the filter were washed with 35 ml of 1% BSA/HBSS buffer. BMVs were immediately collected off the filter with 1% BSA/HBSS and centrifuged at 3000 g for 5 min at 4°C. The supernatant was aspirated and the resulting BMV pellet was frozen and stored at −80°C until further analysis.

### Global Proteomics Using Liquid Chromatography Tandem Mass Spectrometry

Proteomics analysis was performed to quantify the expression of OCT1, OCT2 and OCT3 in BMVs. BMV samples were lysed with a 100 mM Tris-HCl buffer (pH 7.8) containing 50 mM dithiothreitol and 2% sodium dodecyl sulfate and heated for 5 min at 95°C. The samples where sonicated with a Branson-rod-typesonicator and centrifuged at 14000 g for 10 min. The protein concentration was measured with tryptophan fluorescence assay (34) and 100 μg protein was taken for multi-enzyme digestion filter-aided sample preparation (MED-FASP)(35), where proteins were consecutively digested with LysC and trypsin. The digests were concentrated using a GeneVac EX-2plus and injected using an Ultimate 3000 RSLCnano system and separated on an easy spray C18 reversed phase column (50 cm, ID 75 μm) for 145 min on a water/acetonitrile gradient containing 0.1% formic acid. The eluted peptides were analyzed with a Top15 method (full MS followed by ddMS2 scans) on a Orbitrap Q Exactive HF mass spectrometer (ThermoFisher, Waltham, MA). The data were analyzed on MaxQuant version 1.6.10.43 with the complete human proteome extracted from UniProt (September 2020). The false discovery rate was set as 0.01 and match-between-runs was enabled. For the quantification of protein abundance, the total protein approach (TPA) was used (36) for proteins with razor+unique peptides of three and higher. The mass spectrometry proteomics data have been deposited to the ProteomeXchange Consortium via the PRIDE (37) partner repository.

## Results

### HTS Identified Novel OCT3 Inhibitors

**Fig. 1:**
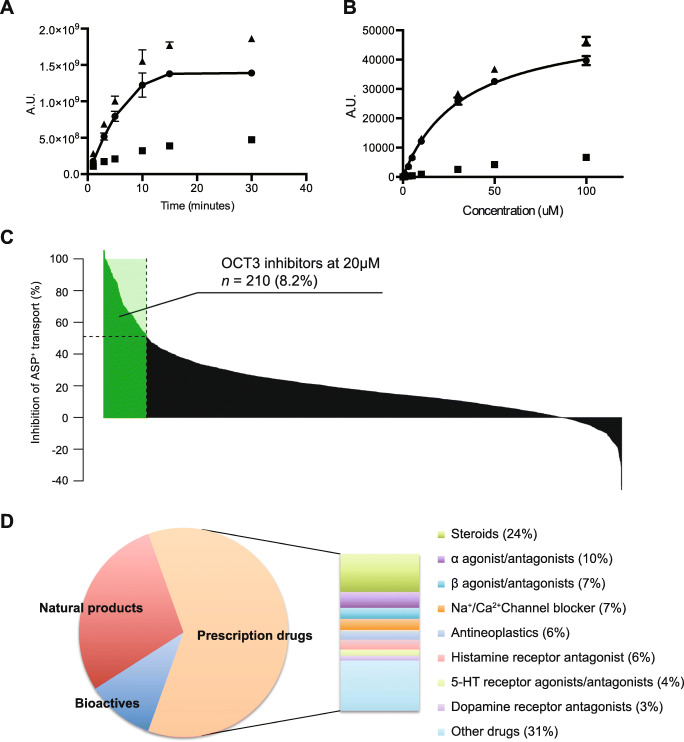
HTS of a 2556 compound library that included prescription drugs and bioactive molecules identified 210 OCT3 inhibitors. (A)Time-dependent ASP^+^ uptake in HEK cells overexpressing OCT3 (▲) or empty vector (■), and OCT3-specific ASP^+^ uptake (●). The uptake was linear for the first 5 min. (B) ASP^+^ initial uptake (at 2 min) increases with concentration in HEK-293 cells stably expressing OCT3. ASP^+^ uptake studies were conducted in HEK cells overexpressing OCT3 (▲) or empty vector (■). Cells were incubated with increasing concentrations of ASP^+^ for 2 min. The K_m_ of OCT3 mediated ASP^+^ uptake was determined to be 33.3 μM (95% CI = 30.5 to 36.1 μM). The uptake kinetic parameters were calculated using the difference in ASP^+^ accumulation between cells overexpressing OCT3 and empty vector cells (●). Data represent mean and 95% confidence intervals, n = 3 per data point. (C) 210 inhibitors capable of inhibiting OCT3 activity by 50% or more at 20 μM were identified among 2556 drugs, natural products, and bioactives. (D) The proportions of the 210 OCT3 inhibitors grouped into drug classes

**Fig. 2:**
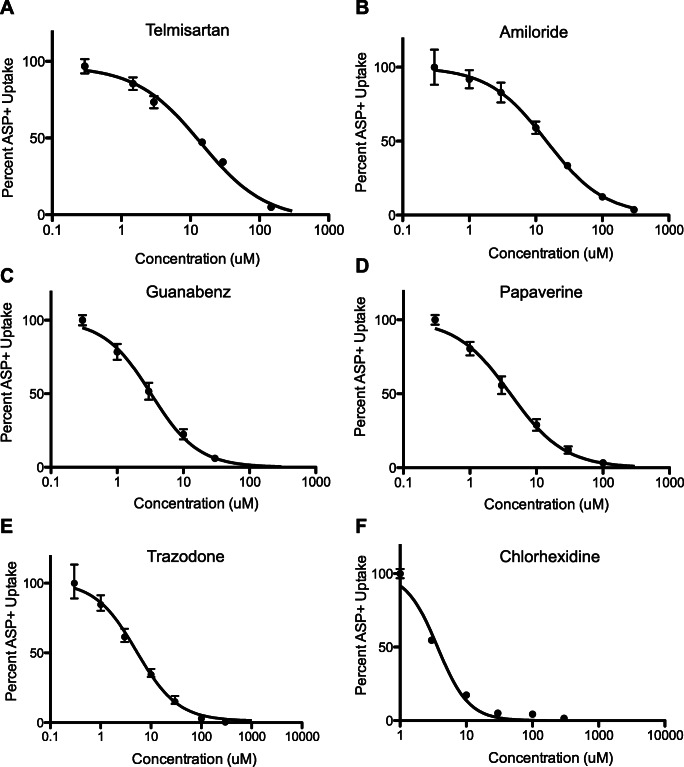
Determination of potency of selected inhibitors of OCT3 against OCT3-mediated ASP^+^ uptake. The concentration of ASP^+^ used in the potency determination was 2 μM. OCT3 inhibitors identified by HTS were validated by determining their IC_50_ values in inhibition studies. (A) The IC_50_ of telmisartan was determined to be 12.0 μM 95% CI = 10.8 to 13.4). (B) The IC_50_ of amiloride was determined to be 14.5 μM (95% CI = 12.9 to 16.4). (C) The IC_50_ of guanabenz was determined to be 3.2 μM (95% CI = 3.0 to 3.6). (D) The IC_50_ of papaverine was determined to be 4.1 μM (95% CI = 3.7 to 4.4). (E) The IC_50_ of trazodone was determined to be 5.2 μM (95% CI = 4.6 to 5.8). (F) The IC_50_ of chlorhexidine was determined to be 3.7 μM (95% CI = 3.3 to 4.1). Data represent mean and 95% confidence intervals, n = 3 per data point

**Fig. 3:**
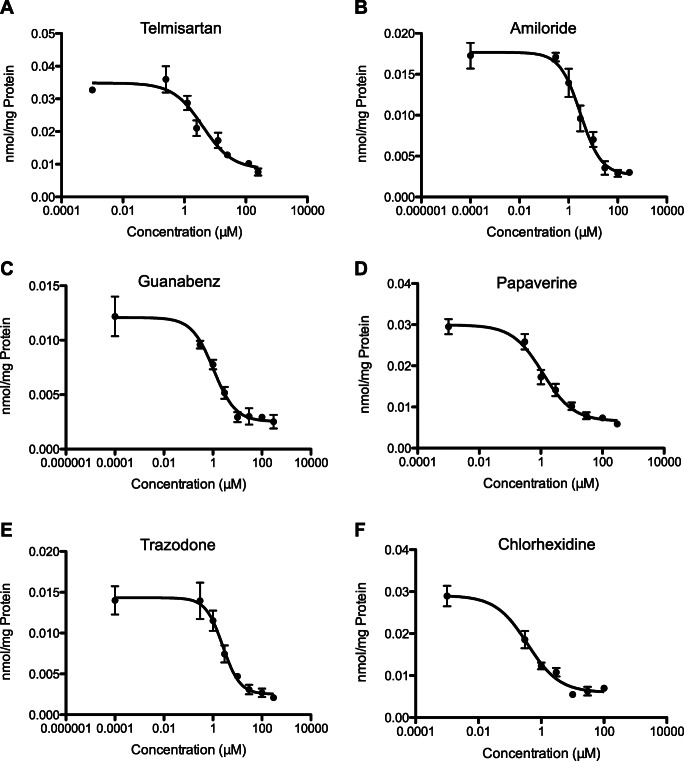
Determination of inhibition potency of selected compounds against OCT3-mediated [^14^C]metformin uptake. The concentration of metformin used in the potency determinations was 1 μM. (A) The IC_50_ of telmisartan was determined to be 3.9 μM (95% CI = 2.4 to 6.2). (B) The IC_50_ of amiloride was determined to be 3.0 μM (95% CI = 2.1 to 4.4). (C) The IC_50_ of guanabenz was determined to be 1.1 μM (95% CI = 0.7 to 1.6). (D) The IC_50_ of papaverine was determined to be 1.2 μM (95% CI = 0.7 to 2.0). (E) The IC_50_ of trazodone was determined to be 2.5 μM (95% CI = 2.0 to 3.4). (F) The IC_50_ of chlorhexidine was determined to be 0.4 μM (95% CI = 0.2 to 0.7). Data represent mean and 95% confidence intervals, n = 3 per data point

A HTS assay was developed using the fluorescent probe, ASP^+^, in HEK cells overexpressing OCT3 as a measurement of transporter activity (24, 28). OCT3 transported ASP^+^ in a time-dependent manner and the uptake was linear until 5 min (Fig. 1A). The K_m_ of OCT3-mediated ASP^+^ uptake was 33.3 μM (95% CI = 30.5 to 36.1 μM, Fig. 1B). Thus, an incubation time of 2 min was used to measure the initial rate of ASP^+^ uptake and an ASP^+^ concentration of 2 μM was used to minimize the effect of substrate concentration on the IC_50_ values (38). In our HTS assay, an inhibitor was defined as any compound that inhibited 50% or more of the ASP^+^ uptake at 20 μM. Of the 2556 compounds in the Spectrum library, we identified 210 (8.2%) OCT3 inhibitors (Fig. 1C). The average Z-prime of the HTS was 0.73, indicating an excellent HTS assay (39). Most inhibitors identified were previously not known to interact with OCT3. Based on estimated IC_50_s, obtained from single-concentration inhibition percentages by assuming a classical sigmoidal concentration-response with a Hill slope of 1, and estimated maximum unbound plasma concentrations, C_U,MAX_, we also predicted that 9 of the 210 inhibitors could potentially cause DDIs (40)(Table I). When we considered total maximum plasma concentrations instead, a total of 23 compounds were identified to potentially inhibit OCT3 (Table I). Further inhibition studies for many of the 23 compounds were conducted to validate the screen, and the results reaffirm the predicted IC_50_(Figs. 2, 3, and Table SI).

**Table I:** Prescription drugs that are predicted to inhibit OCT3 at clinically relevant plasma concentrations

Compound	Inhibition (%)	Predicted IC_50_ (μM)*	C_MAX_(μM) †	Protein binding	C_MAX_/ IC_50_	Unbound C_MAX_/ IC_50_
Compounds with unbound C_MAX_/ IC_50_ > 0.1						
azlocillin	66.9	9.9	17.3	30%	1.75	1.23
aztreonam	87.1	5	585	56%	117	51.5
famotidine	66.9	9.9	308	10%	31.1	28.0
flufenamic acid	60.6	13.0	24.2	90%	1.9	0.19
meropenem	65	10.8	256	2%	23.8	23.3
propafenone	74	7.0	7.9	85%	1.13	0.17
quinine	90.6	5	10.7	69%	2.15	0.67
trazodone	94.5	5	7.6	90%	1.53	0.15
trimethoprim	59.3	13.7	5.9	44%	0.43	0.24
Compounds with total C_MAX_/ IC_50_ > 0.1						
cilostazol	93.8	5	2.1	95%	0.42	
emetine	82.7	5	0.6	90%	0.12	
exemestane	94.5	5	1.4	90%	0.28	
glimepiride	87.7	5	1.2	99%	0.24	
imatinib	72.2	7.7	1.2	95%	0.16	
ketoconazole	93.4	5	1.9	91%	0.38	
lansoprazole	87.2	5	2.9	97%	0.59	
leflunomide	50.5	19.6	233	99.3%	11.9	
omeprazole	64.1	11.2	4.2	95%	0.38	
prednisolone	67.5	9.6	2.9	70%	0.30	
rabeprazole	87.3	5	1.3	96%	0.26	
spironolactone	82.8	5	1.1	93%	0.22	
telmisartan	81.6	5	2.8	99.5%	0.56	
valdecoxib	53.6	17.3	2.2	98%	0.13	

*Predicted IC_50_ is calculated based on a single data point reflecting the percent inhibition of OCT3 at the screening concentration of the inhibitor (Materials and Methods)

† C_MAX_ values were obtained from http://www.micromedexsolutions.com/

The 210 inhibitors identified in the screen were grouped into pharmacological classes (Fig. 1D). Similar to inhibitors of OCT1, steroids, antihistamines, and α-adrenergic receptor antagonists were more likely to inhibit OCT3 (24). In addition, we identified OCT3 inhibitors in other classes as well, including β-adrenergic receptor agonists/antagonists, sodium/calcium channel blockers, 5-HT receptor agonists/antagonists, and dopamine receptor agonists/antagonists. However, tricyclic antidepressants (TCAs), which are inhibitors of OCT1 and OCT2 (24, 28), were not enriched in our HTS. In fact, none of the eight TCAs tested inhibited OCT3 mediated ASP^+^ uptake at 20 μM by 50% or more.

Selected compounds previously not known to inhibit OCT3 (telmisartan, amiloride, guanabenz, papaverine, trazodone and chlorhexidine) were validated by determining their IC_50_ in inhibition studies (Fig. 2). All six compounds had IC_50_ values below 20 μM when tested against OCT3 mediated ASP^+^ uptake. Interestingly, when the same six compounds were tested as inhibitors of OCT3-mediated [^14^C]metformin uptake, the IC_50_ values were generally lower than those of ASP^+^ uptake (Fig. 3). For example, the IC_50_ values against ASP^+^ uptake for telmisartan and amiloride were determined to be 12.0 μM (95% CI = 10.8 to 13.4) and 14.5 μM (95% CI = 12.9 to 16.4), respectively (Fig. 2A, B). When tested against OCT3 mediated [^14^C]metformin uptake, the IC_50_ values for telmisartan and amiloride were determined to be 3.9 μM (95% CI = 2.4 to 6.2) and 3.0 μM, respectively (Fig. 3A, B). Of note, substrate concentrations of both ASP^+^ and metformin were well below their K_m_ values (see Materials and Methods), so the IC_50_ values obtained are equivalent to their K_i_ values. The result suggests that the kinetics of inhibition of OCT3-mediated uptake may be substrate-dependent.

### Structure-Activity Relationship, SAR, Modeling

**Fig. 4:**
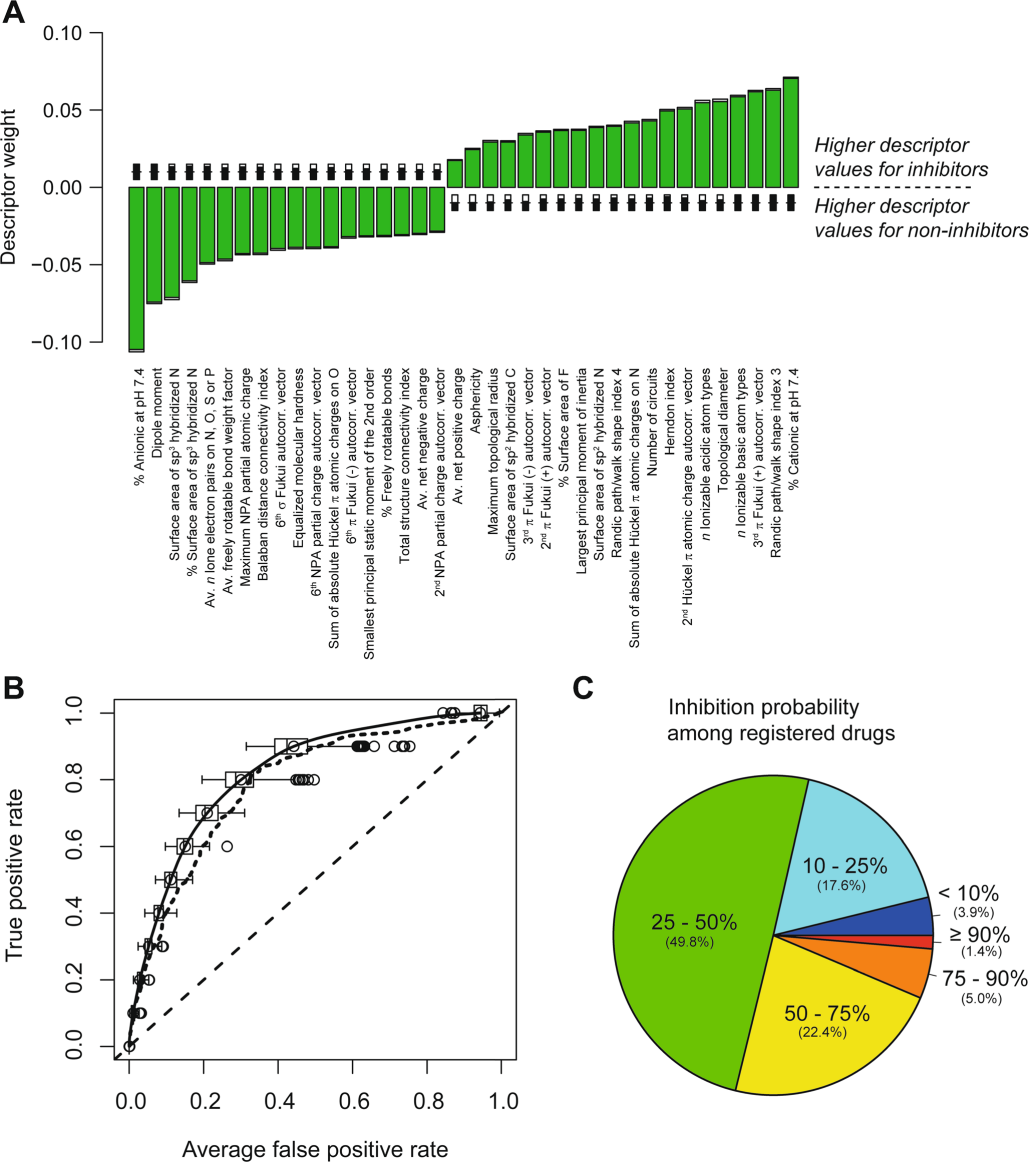
SAR model and virtual screening against the DrugBank database of registered small-molecule drugs. (A) Important molecular descriptors used to classify OCT3 inhibitors and noninhibitors. Bar shows the mean PLS regression coefficients from cross-validated models. Descriptors with positive coefficient have higher values in inhibitors, and descriptors with negative values have higher values in noninhibitors. (B) ROC curves for 100 retrospective cross-validation runs. Average ROC curve is shown in solid black line. The AUC for the final SAR model was determined to be 0.77. (C) Inhibition probability result from the virtual screen against registered drugs. Percentage ranges (larger font) indicate predicted probabilities of belonging to the inhibitor class. Numbers in parentheses indicate the fraction of all predictions falling into a particular probability range

Next, we generated a SAR model capable of discriminating OCT3 inhibitors from noninhibitors. The 2556 compounds were first classified as inhibitors or noninhibitors of OCT3 based on their ability to inhibit >50% of ASP^+^ uptake at 20 μM. Molecular descriptors were generated for each compound and were used to develop the model. Using PLS projection, we were able to identify descriptors that could classify inhibitors and noninhibitors. After optimization through an iterative variable procedure (i.e. descriptors with low influence on the model were removed in a step-wise manner, removing smaller and smaller chunks as the selection progressed), we arrived at a set of important descriptors used in the final model (Fig. 4A). As expected, cationic compounds were more likely to interact with OCT3. Inhibitors are also more likely to be larger (topological diameter), spherical (asphericity), and with less freely rotatable bounds. Lastly, a double-loop cross-validation procedure was used to evaluate the SAR model, since this gives an unbiased estimate of external model predictivity (Table II). The final SAR model had an average accuracy of 0.76, and an average precision of 0.58. The receiver operating characteristic (ROC) curve (Fig. 4B), a graphical representation of the performance of a model, showed an area under the curve (AUC) of 0.77, indicating a good binary classifier SAR model.

**Table II:** Statistics of the derived SAR model for inhibitors of OCT3

	Internal (confidence interval)		External (confidence interval)	
n components	4.4 ± 0.1	1–11		
n variables	45.5 ± 0.6	10–73		
AUC	0.82 ± 0.0008	0.75–0.86	0.77 ± 0.0008	0.75–0.79
MCC	0.32 ± 0.0011	0.23–0.40	0.25 ± 0.0015	0.21–0.28
Accuracy	0.78 ± 0.0008	0.72–0.83	0.76 ± 0.0008	0.74–0.78
Balanced accuracy	0.75 ± 0.0007	0.68–0.80	0.70 ± 0.0011	0.67–0.72
Informedness	0.50 ± 0.0014	0.36–0.61	0.40 ± 0.0023	0.33–0.45
TPR* precision	0.23 ± 0.0008	0.18–0.29	0.20 ± 0.0008	0.18–0.22
TNR* precision	0.97 ± 0.0001	0.95–0.98	0.96 ± 0.0002	0.95–0.96
TPR* recall	0.72 ± 0.0010	0.59–0.81	0.62 ± 0.0023	0.56–0.68
TNR* recall	0.78 ± 0.0008	0.73–0.84	0.77 ± 0.0008	0.75–0.80
Average precision	0.60 ± 0.0001	0.18–0.98	0.58 ± 0.0003	0.18–0.96
Average recall	0.75 ± 0.0001	0.59–0.84	0.70 ± 0.0013	0.56–0.80

*True positive rate (TPR); true negative rate (TNR)

### Virtual Screening by SAR Model of a Drug Library

**Fig. 5:**
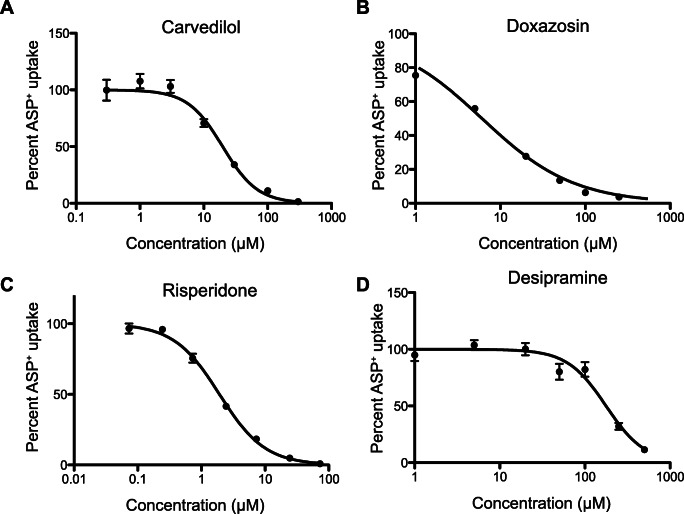
In vitro validation of OCT3 inhibitors identified through use of the SAR model. ASP^+^ (2 uM) was used as substrate. (A) SAR model predicted carvedilol has an 86% probability of inhibiting OCT3. The IC_50_ was determined to be 19.7 μM (95% CI = 17.5 to 22.2). (B) SAR model predicted doxazosin has a 93% probability of inhibiting OCT3. The IC_50_ was determined to be 5.6 μM (95% CI = 5.0 to 6.2). (C) SAR model predicted risperidone has an 82% probability of inhibiting OCT3. The IC_50_ was determined to be 1.9 μM (95% CI = 1.7 to 2.1). (D) SAR model predicted desipramine has a 55% probability of inhibiting OCT3 (below 75% cutoff). The IC_50_ was determined to be 177 μM (95% CI = 148.6 to 211.0). Data represent mean ± SD, *n* = 6 per data point

In this study, we screened a large compound library, the Spectrum library, for OCT3 inhibitors. While the library contains 2556 compounds, only 60% of the compounds are drugs and the rest are bioactives and natural products. In order to identify additional OCT3 inhibitors among registered drugs, we applied our SAR model against 2643 registered drugs in the DrugBank database to predict OCT3 inhibitors in silico. Using the SAR model, each drug in the database was assigned a probability value of interacting with OCT3 (Fig. 4C). Enrichment of known OCT3 inhibitors were 13-fold, 7-fold and 6-fold within the top 1, 5 and 10% of the predictions among registered drugs (Fig. SI), with known OCT3 inhibitors such as the tyrosine kinase inhibitors imatinib and erlotinib recovered in the top 0.1 and 6% of the hit list, respectively. Importantly, enrichment factors in databases like the registered drug collection used here are likely underestimated, assuming that the actual fraction of OCT3-inhibiting compounds reflect the hit rate in our screening of the Spectrum library (8.2%). Using 75% as the cutoff inhibition probability, 6.4% of the 2643 compounds were predicted to interact with OCT3; many of these drugs were novel OCT3 inhibitors and were not included in Spectrum library. Selected compounds were validated by determining their IC_50_ in inhibition studies. From the in silico model, carvedilol, doxazosin, and risperidone had probabilities of 86%, 93%, and 82%, respectively, of inhibiting OCT3. Experimental studies confirmed the predictions, and the IC_50_ values of the three compounds were determined to be 19.7 μM, 5.6 μM, and 1.9 μM, respectively (Fig. 5A, B, C). In contrast, desipramine had a probability of 55% of inhibiting OCT3, below the cutoff value, and did not inhibit 50% or more OCT3 activity at 20 μM (Fig. 5D).

### Proteomic and Transcriptomic Expression Levels of OCTs in Human Brain Microvessels (BMVs)

**Fig. 6:**
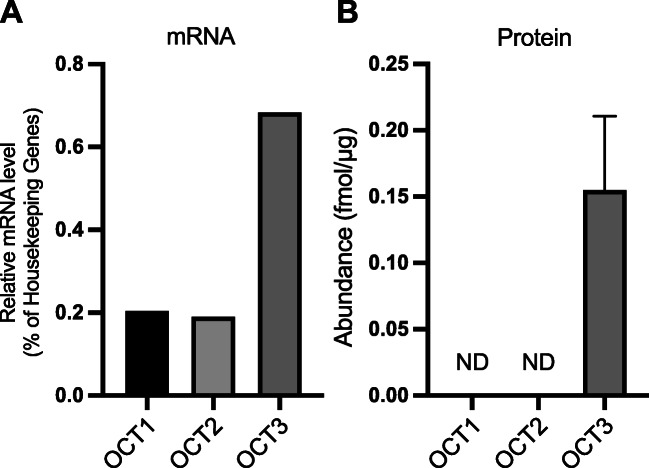
Transcriptomic and proteomic expression levels of OCTs in human brain microvessels (BMVs). (A) mRNA level of OCT1, OCT2 and OCT3 relative to the mean of three housekeeping genes: GAPDH, β-actin, and β2 microglobulin. Data from n = 2 (Plot generated from data from Geier et al., 2013). (B) Protein expression abundance of OCTs in BMVs. The abundance of OCT3 was 0.155 ± 0.056 fmol/μg total protein, OCT1 and OCT2 were not detectable in our proteomic study. Data represent mean ± SEM, n = 5

OCT3 is a transporter with pleiotropic effects including important neuropsychiatric effects. It is not known whether OCT3 or its paralogs, OCT1 or OCT2, are expressed in the BBB and play a role in the entry of drugs and other compounds into the CNS. Accordingly, we performed proteomic analysis on adult human BMVs to measure the levels of OCT1, OCT2 and OCT3 at the BBB. We have previously reported that the mRNA level of OCT3 was higher than OCT1 and OCT2 in BMVs isolated from 2 healthy adult donors (41) (Fig. 6A). Consistent with the transcriptomic data, OCT3 was the most highly expressed OCT (0.155 ± 0.056 fmol/μg total protein) in the BBB in our proteomic study, as OCT1 and OCT2 proteins were not detected (Fig. 6B). We also detected other transporters previously reported in the human BBB, such as SLC7A5 and SLCO2B1, which were measured at 2.03 ± 0.573 and 0.227 ± 0.081 fmol/μg total protein, respectively. These data suggest that OCT3 may indeed be an important transporter for entry of many basic drugs into the CNS.

## Discussion

OCT3 is a transporter ubiquitously expressed in many tissues including tissues of pharmacological interest such as the brain, liver, kidney, and intestine. Animal experiments with Oct3 knockout mice and genetic associations studies of polymorphisms in OCT3 have revealed that the transporter is associated with a range of effects including effects on the CNS (42), cardiovascular disease (9, 10, 43), and cancer (44). Further, studies suggest that the transporter may have an important role in the pharmacokinetics of many drugs; however, its role in pharmacokinetics and DDIs has been poorly characterized. The goals of this study were to conduct a HTS of a prescription drug library to identify OCT3 inhibitors and to develop a predictive SAR model capable of distinguishing between inhibitors and noninhibitors of OCT3. Our key findings are: (a) OCT3 is highly druggable; about 10% of the compounds screened are inhibitors of the transporter; (b) several drugs are capable of inhibiting OCT3 at clinically relevant unbound concentrations; (c) an SAR model was developed to predict OCT3 inhibitors; and (d) proteomic studies reveal that OCT3 is the most highly expressed OCT in the human BBB. Each of these findings is discussed below.

### OCT3 Is Highly Druggable

Our HTS of a large compound library consisting of 2556 prescription drugs, bioactive, and natural products, identified 210 inhibitors that inhibit 50% or more of OCT3-mediated uptake at 20 μM (Fig. 1C). In recent studies, approximately 25 tyrosine kinase inhibitors have been screened for inhibition of OCT3 (45, 46); however, our study is the first large-scale inhibitor screen against OCT3. Most of the inhibitors identified were novel, i.e., not previously known to interact with OCT3. Certain drug classes were more likely to contain inhibitors of OCT3 including steroids and adrenoreceptor agonists and antagonists (Fig. 1D). Drugs in several of the classes have also been found to inhibit OCT1 and OCT2 (28, 29, 47). Surprisingly, TCAs, many of which are well-established inhibitors of OCT1 and OCT2, were not included among the classes of drugs that are enriched for inhibitors of OCT3. Notably, none of the TCAs tested were inhibitors of OCT3. This observation may be explained by structural differences in the substrate binding pocket among the OCTs. While OCT1 and OCT2 share a higher degree of homology including many residues predicted to be important in substrate binding, OCT3 differs significantly in both its homology and key residues (26). To date, high resolution crystal structures of OCTs are not available.

### Several Drugs Are Capable of Inhibiting OCT3 at Clinically Relevant Unbound Concentrations

Based on the predicted IC_50_ values, we identified nine drugs with unbound plasma concentrations greater than 0.1 times their IC_50_ values after therapeutic doses, and an additional 14 drugs that had total plasma concentrations greater than 0.1 times their IC_50_ values (Table I). These data suggest that the nine compounds interact with OCT3 at clinically relevant unbound concentrations and as such, have the potential to cause clinical DDIs with metformin or other substrates of OCT3 including fenoterol, sumatriptan, lamuvidine and trospium. Multiple human genetic studies have replicated associations of polymorphisms in SLC22A3 with coronary artery diseases (9, 10, 43) including recent studies in knockout mice (46). Notably, five of the nine drugs predicted to interact with OCT3 at clinically relevant unbound concentrations have warnings and precautions related to cardiovascular side-effects (e.g. cardiac arrhythmia) (Table I, Table SII). These five FDA approved drugs are aztreonam, propafenone, quinine, trazodone, and trimethoprim. Though speculative, it is possible that inhibition of OCT3 may lead to some of these side effects. Clearly, further research is needed. Of note is that the compounds identified in this study as potential clinically relevant inhibitors of OCT3 (Table I) did not overlap with the clinically relevant inhibitors identified in a prescription drug library screen of OCT2 (disopyramide, dipyridamole, imipramine, tacrine, orphenadrine, ondansetron, and cimetidine) (28). However, trimethoprim has been shown previously to be an inhibitor of OCTs and MATEs and to reduce the renal secretion of zidovudine (which may involve OCT2) though the mechanism is not clear (29, 48). Thus, it may be possible to use one of the inhibitors (e.g. meropenam and aztreonam) as an isoform specific inhibitor in in vivo studies to examine the role of OCT3 in drug absorption, disposition and response.

### An SAR Model Was Developed to Predict OCT3 Inhibitors

In light of the inclusion of transporters in the FDA draft guidance on drug interactions, increasingly, the requirements to identify transporter inhibitors and substrates will be expanded. Predictive models for both substrates and inhibitors are needed to inform in vitro studies carried out during drug discovery and development to assess DDI liabilities. Here, we developed an SAR model using data generated by our HTS. The predictive SAR model is capable of identifying OCT3 inhibitors and noninhibitors (Fig. 4). A virtual screen using the SAR model against current registered drugs showed that our SAR model was able to accurately identify known inhibitors (Fig. SI) and predict novel OCT3 inhibitors (Figs. 4 and 5). Until recently, it was often assumed that transporter inhibitors interact with the protein in a competitive manner only. Studies now suggest that the inhibition can occur by other mechanisms as well (competitive inhibition, non-competitive inhibition, mixed inhibition) (49). This is especially true for polyspecific transporters, where multiple substrate binding sites may exist. The International Transporter Consortium recently acknowledged that our lack of understanding of the inhibition mechanism is a limiting factor in transporter studies (50). However, our HTS and SAR model would not be able to identify or predict the mechanism of inhibition.

Though no high resolution structures are available for any mammalian OCT, or for that matter, for any human SLC22 transporter, a prokaryotic homolog of the SLC family protein, LeuT, has been used to generate comparative models of human SLC transporters (51–53). However, its low sequence similarity to OCTs casts doubt on comparative models generated based on LeuT structure. New technology, such as the cryo-transmission electron microscopy, is promising in solving membrane transporter structures and has been successfully used to study the interactions between human SLCs and substrates or inhibitors in their binding sites (54, 55). Future studies are needed to explore these options to generate structure-based model for polyspecific transporters. In addition to extended profiling of alternative ligand binding mechanisms, using multiple substrates and/or substrate concentrations, such structural models can provide important insight into binding modes and substrate-selective inhibition mechanisms.

### Proteomic Studies Reveal that OCT3 is the Most Highly Expressed OCT in the Human BBB

Our proteomic analysis detected OCT3 protein expression in BMVs isolated from the insular cortex while expression of other OCTs were not detected. Proteomics studies of human BBB have reported conflicting results regarding the expression levels of OCT proteins. OCT3 was under the limit of quantification in studies by Uchida et al. and Shawahna et al., however, OCT3 was the highest expressed OCT in a study by Al-Majdoub et al. (56–58). Our results are in agreement with those of Al-Majdoub et al. Differences among studies may be due to different sample preparation methods as well as different methods used in the proteomics studies, e.g., targeted versus global proteomics (59). The use of different brain regions for sample preparation may also explain some of the differences in reported values. Our results are potentially relevant to clinical DDIs in the BBB. For example, the nine OCT3 inhibitors may potentially inhibit OCT3 in the BBB (or elsewhere) at clinically relevant unbound concentrations. Hypothetically, these compounds may reduce the brain penetration of other drugs that rely on OCT3 to enter the CNS. Further, several of the nine compounds (e.g. aztreonam, famotidine, meropenem and trimethoprim) have neurological side effects (Table SII), which though speculative, may reflect inhibition of OCT3 in the brain (60). However, as noted, none of the TCAs were good inhibitors of OCT3, suggesting that inhibition of OCT3 may not be a mechanism for either the effects or side-effects associated with TCAs. Future studies are needed to explore the pharmacological impact of OCT3 inhibitors.

## Conclusion

In conclusion, we developed and conducted a HTS against a large compound library to identify OCT3 inhibitors. Our results suggest that the transporter interacts with many prescription drugs, most of which were not previously known to interact with OCT3. Several of the drugs can potentially inhibit OCT3 at clinically relevant drug concentrations. Further we determined that OCT3 is the most highly expressed OCT at the human BBB which suggests that the transporter may be involved in the entry of a diverse array of substrates into the CNS. Further studies need to be conducted to determine whether the clinically relevant inhibitors identified in this study may perpetrate off-target effects mediated by OCT3 or DDIs with OCT3 substrates. We hope that this study, which included proteomic studies of OCT3 in the human BBB, as well as new ligands for this transporter has added to the body of work of Professor Emerita Margareta Hammarlund-Udenaes who is a pioneer in BBB research in the pharmaceutical sciences (61–63), and to the concepts and methodologies in improving treatment of neurological diseases (64). Future research is needed to understand the role of organic cation transporters in the pharmacokinetics and pharmacodynamics of drugs in the brain.

## Supplementary Information

Fig. S1 (PNG 43.7 kb)

High Resolution Image (EPS 1113 kb)

ESM 2 (DOCX 17.9 kb)

ESM 3 (XLSX 13 kb)
