# Supplementary figures and images for: High Throughput Screening of a Prescription Drug Library for Inhibitors of Organic Cation Transporter 3, OCT3
Source: Pharm Res. 2022 Jan 28;39(7):1599–613. doi: 10.1007/s11095-022-03171-8 (PMC9246766; doi:10.1007/s11095-022-03171-8)

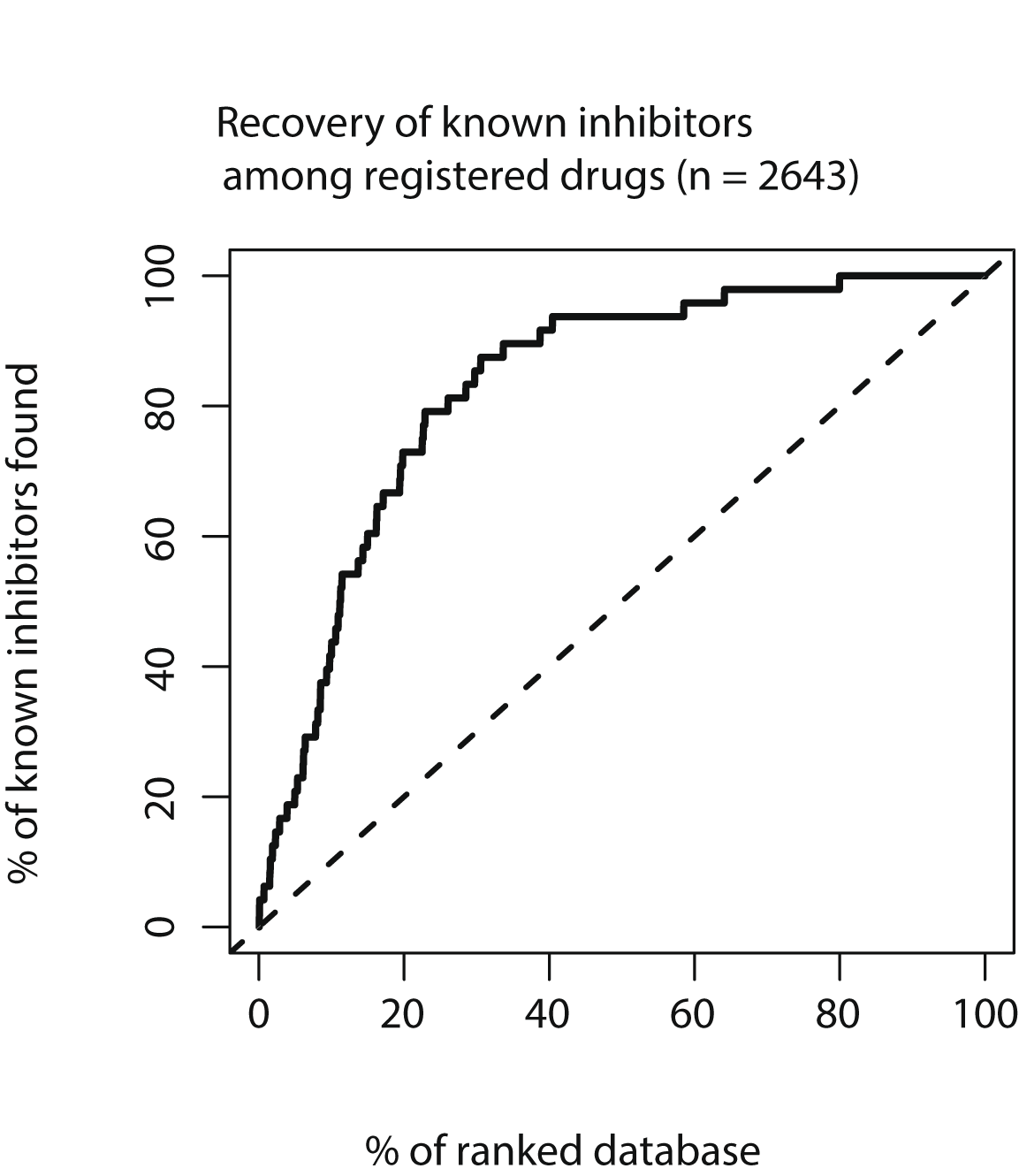

Supplement: Supplementary file 1 — (PNG 43.7 kb) [file 11095_2022_3171_Fig7_ESM.png]
