# Supplementary material for: High Throughput Screening of a Prescription Drug Library for Inhibitors of Organic Cation Transporter 3, OCT3
Source: Pharm Res. 2022 Jan 28;39(7):1599–613. doi: 10.1007/s11095-022-03171-8 (PMC9246766; doi:10.1007/s11095-022-03171-8)
Supplement: Supplementary file 3 — (DOCX 17.9 kb) [file 11095_2022_3171_MOESM2_ESM.docx]

| Compound | | Inhibition (%) | Predicted IC­­_50_ (μM)* | Experimental IC­­_50_ (μM) |
| --- | --- | --- | --- | --- |
|  | |  |  |  |
|  | Compounds with unbound C_MAX_/ IC­­_50_ > 0.1 | | | |
|  |  |  |  |  |
| famotidine | | 66.9 | 9.9 | 6.7±2.0 (ref. 1) |
| propafenone | | 74 | 7.0 | 2.4 (2.1-2.7) |
| quinine | | 90.6 | 5 | 6.6 (6.1-7.3) |
| trazodone | | 94.5 | 5 | 5.2 (4.6-5.8) |
| trimethoprim | | 59.3 | 13.7 | 12.3±5.2 (ref. 2) |
|  | |  |  |  |
|  | Compounds with total C_MAX_/ IC­­_50_ > 0.1 | | | |
|  | |  |  |  |
| imatinib | | 72.2 | 7.7 | 4.4±1.1 (ref. 3) |
| ketoconazole | | 93.4 | 5 | 0.42 (0.38-0.46) |
| lansoprazole | | 87.2 | 5 | 3.1±1.3 (ref. 4) |
| omeprazole | | 64.1 | 11.2 | 22±2.2 (ref. 4) |
| rabeprazole | | 87.3 | 5 | 3.0±0.8 (ref. 4) |
| telmisartan | | 81.6 | 5 | 12 (10.8-13.4) |
|  | |  |  |  |
|  | |  |  |  |

**Supplemental Table I. IC_50_ values of selected prescription drugs that are predicted to inhibit OCT3 at clinically relevant plasma concentrations**

IC_50_ values are determined in-house unless otherwise referenced. For the in-house generated data, the values represent mean and 95% confidence intervals, n = 6 per data point.

1. Bourdet, D. L., Pritchard, J. B. & Thakker, D. R. Differential Substrate and Inhibitory Activities of Ranitidine and Famotidine toward Human Organic Cation Transporter 1 (hOCT1; SLC22A1), hOCT2 (SLC22A2), and hOCT3 (SLC22A3). *J Pharmacol Exp Ther* **315**, 1288–1297 (2005).
2. Lepist, E.-I. *et al.* Contribution of the organic anion transporter OAT2 to the renal active tubular secretion of creatinine and mechanism for serum creatinine elevations caused by cobicistat. *Kidney Int* **86**, 350–357 (2014).
3. Minematsu, T. & Giacomini, K. M. Interactions of Tyrosine Kinase Inhibitors with Organic Cation Transporters and Multidrug and Toxic Compound Extrusion Proteins. *Mol Cancer Ther* **10**, 531–539 (2011).
4. Nies, A. T. *et al.* Proton pump inhibitors inhibit metformin uptake by organic cation transporters (OCTs). *Plos One* **6**, 1--11 (2011).
